# Development and characterization of western corn rootworm (*Diabrotica virgifera virgifera* LeConte) resistant to non-Cry insecticidal proteins Mpp75Aa1.1 and Vpb4Da2

**DOI:** 10.1371/journal.pone.0359428

**Published:** 2026-11-02

**Authors:** William Moar, Chitvan Khajuria, Kaylee Miller, Michael Pleau, Brent Werner, Paula Price, Jennifer Fridley, Yong Yin, Alan Willse, Andrew J. Tyre, Graham Head

**Affiliations:** Bayer Crop Science, Chesterfield, Missouri, United States of America; Assam Agricultural University Faculty of Agriculture, INDIA

## Abstract

Transgenic maize expressing *Bacillus thuringiensis* (Bt) proteins, and more recently DvSnf7 dsRNA, has been adopted across the Midwestern U.S. Corn Belt to control western corn rootworm (WCR; *Diabrotica virgifera virgifera* LeConte) and other corn rootworm species. However, resistance has been reported to all five Bt proteins currently used to control WCR. This leaves DvSnf7 dsRNA as the only trait active against WCR without reported field resistance, emphasizing the need for alternative management tools. Mpp75Aa1.1 from *Brevibacillus laterosporus* and Vpb4Da2 from Bt are two proteins with high efficacy against WCR that do not have cross-resistance to any current commercial Bt protein. To predict the nature of future WCR field resistance to these proteins when used *in planta*, WCR colonies resistant to maize plants expressing either Mpp75Aa1.1 or Vpb4Da2 were developed by repeated selection on plants containing that protein. We confirmed resistance for both the Mpp75Aa1.1-selected colony (after generation 13, with 10 under selection) and the Vpb4Da2-selected colony (after generation 14, with 10 under selection) using larval recovery and beetle emergence assays with insecticidal protein–expressing maize seedlings and protein diet bioassays. Mpp75Aa1.1-resistant WCR survived and emerged comparably on Mpp75Aa1.1-expressing and non-traited maize, and exhibited a concentration response with an estimated resistance ratio of 5 on diet containing Mpp75Aa1.1. Vpb4Da2-resistant WCR also survived and emerged comparably on Vpb4Da2-expressing and non-traited maize and had no concentration response (i.e., no mortality) on diet containing Vpb4Da2, indicating a high level of resistance. Dominance for both resistant WCR colonies ranged from semidominant to incompletely recessive with increasing dose; effective dominance was estimated to be 0.23 (95% CI 0.12–0.35) and 0.24 (0.16–0.32) for Mpp75Aa1.1 and Vpb4Da2, respectively. These resistant colonies will be a useful tool for understanding the risk of WCR evolving resistance to these proteins when they are deployed commercially in maize.

## Introduction

Transgenic maize expressing *Bacillus thuringiensis* (Bt) proteins, and more recently DvSnf7 dsRNA, has been rapidly adopted on farms across the Midwestern U.S. Corn Belt to control western corn rootworm (WCR; *Diabrotica virgifera virgifera* LeConte) and other corn rootworm (CRW; *Diabrotica* spp. [Coleoptera]) species [1,2]. However, at least some resistance has been reported to all five Bt proteins currently used to control WCR [3], leaving DvSnf7 dsRNA as the only plant-incorporated protectant without reported resistance and emphasizing the need for alternative management tools. Numerous new proteins have been described that control CRW, including from Bt [4], *Brevibacillus laterosporus* [5], *Alcaligenes faecalis* [6]*, Chromobacterium piscinae* [7], *Pleurotus* spp [8]., *Pseudomonas chlororaphis* [9], and *Ophioglossum pendulum* [10]. Two such proteins, Mpp75Aa1.1 from *B. laterosporus* and Vpb4Da2 from Bt, have high efficacy against WCR and no cross-resistance to any current commercial Bt protein [4,5]. Both proteins have modes of action similar to those of the well-characterized Bt Cry proteins and therefore have attractive safety profiles for use *in planta* [11,12].

To predict the nature of future WCR field resistance to plant-incorporated Mpp75Aa1.1 and Vpb4Da2, we developed WCR colonies resistant to maize plants expressing each protein. This report describes the development of these two colonies and provides data on the level and dominance of resistance in each one. These results provide the first information on WCR resistance to these two new pore-forming proteins [13].

## Materials and methods

### Establishing Mpp75Aa1.1- and Vpb4Da2-resistant WCR colonies

In 2018, seeds of maize expressing Mpp75Aa1.1 (hereafter Mpp75Aa1.1 maize), Vpb4Da2 (Vpb4Da2 maize), or neither trait (non-traited [NT] maize) were planted in large and small plots located in Pilger and Leigh, NE, USA, and Waterman, IL, USA, where previous observations indicated relatively high WCR egg numbers [14]. The Mpp75Aa1.1 and Vpb4Da2 maize events were previously shown to produce doses (here defined as percent reduction in adult WCR emergence compared to that of NT maize) of 99% and 97%, respectively [4,5]. For all plots, plants were checked for expression of the expected trait using Bayer’s Molecular Quality Characterization (MQC) testing protocols, which use event-specific PCR analysis [14]. After removal of plants that did not test as expected, there were approximately 3300, 3400, and 270 Mpp75Aa1.1, Vpb4Da2, and NT plants, respectively, across the NE sites and approximately 2700, 2800, and 270 Mpp75Aa1.1, Vpb4Da2, and NT plants, respectively, from the IL site. Approximately eight weeks after planting, before tasseling, the tops of the plants were cut off above the third or fourth green healthy leaf, approximately 40–60 cm from the soil surface, to allow tenting. Large tents (e.g., 40 ft × 90 ft) and small tents (e.g., 12 ft × 12 ft) were erected over large and small plots, respectively. CRW beetles from the NE and IL sites were collected at least once per week and transported to the University of Nebraska–Lincoln and the Bayer CropScience (Waterman, IL) insectary, respectively, for rearing and selection. In NE, a total of 666, 734, and 4082 CRW beetles were collected from Mpp75Aa1.1, Vpb4Da2, and NT maize, respectively. In IL, a total of 118, 104, and 1004 CRW beetles were collected from Mpp75Aa1.1, Vpb4Da2, and NT maize, respectively. On the same day CRW beetles were collected from the field, they were brought into the lab and separated by sex and species (WCR, northern corn rootworm [NCR; *Diabrotica barberi*], and southern corn rootworm [SCR; *Diabrotica undecimpunctata*]).

Tent-collected WCR males from the NE and IL populations were crossed with non-diapausing (ND) virgin females from a susceptible lab-reared colony (Waterman non-diapausing colony) at the University of Nebraska–Lincoln and Bayer Crop Science (Waterman, IL), respectively. All adult oviposition cages were provided with a proprietary artificial diet and placed under conditions to optimize oviposition: 23.8°C, 45% relative humidity (RH), and 14:10 hours light:dark [14]. The number of ND females crossed with tent-collected males varied based on availability of ND females on the day of collection. In total, 48 WCR males from the Mpp75Aa1.1 tents were crossed with 72 ND virgin females, 41 WCR males from Vpb4Da2 maize tents were crossed with 151 ND virgin females, and 326 WCR male beetles from the NT maize tents were crossed with 491 ND virgin females. These crosses produced an Mpp75Aa1.1-resistant colony, a Vpb4Da2-resistant colony, and a susceptible control colony, respectively. Tent-collected females were separated based on gravidity. Gravid females were provided with food and oviposition dishes to collect diapausing eggs. These eggs were collected twice per week and stored at 8°C for up to 40 weeks in case F2 larvae from mating of tent-collected males with ND females did not survive when placed on traited plants. Between 500 and 600 F1 eggs from tent-collected males crossed with the ND females were infested per tray (Dart brand “Clear Seal” 21 × 21 × 7.6 cm) containing ca. 160 NT maize seedlings in 400 mL of soil and 40 mL of water, producing 18, 50, and 194 trays for Mpp75Aa1.1-resistant, Vpb4Da2-resistant, and susceptible WCR, respectively. All root mat trays for larval development (and selection) were placed under the following environmental settings: 25°C, 45% RH, and 0:24 hours light:dark. After the initiation of pupation, trays were placed in emergence cages and beetles collected under the following environmental settings: 25°C, 45% RH, and 24:0 hours light:dark. Beetles were sib-mated to produce F2 eggs (see S1 and S2 Figs). At F2, neonates from all locations for each colony were combined at the time of infestation for selection on Mpp75Aa1.1 or Vpb4Da2 maize seedlings (as described above). After this first round of selection, nine beetles survived from the Mpp75Aa1.1-resistant colony and 309 beetles survived from the Vpb4Da2-resistant colony.

The nine surviving F2 individuals from the Mpp75Aa1.1-resistant colony did not produce sufficient eggs to continue the colony. Therefore, in early 2019, diapausing eggs that were collected from Mpp75Aa1.1 tent-collected females in 2018 from the NE and IL locations were broken out of diapause (by exposing the eggs to 25°C for approximately 10 days) and combined at the time of infestation on NT maize. Emerging males were crossed with ND females. F1 and F2 larvae were fed NT maize roots. F3 neonates were selected on Mpp75Aa1.1 maize. Only 10 individuals survived this first round of selection, but they produced sufficient eggs to continue the colony. F4 larvae were relaxed (fed NT maize), and F5–F13 were selected on Mpp75Aa1.1 maize (for a total of 13 generations of which 10 were under selection; see S1 Fig).

The 309 surviving F2 individuals from the Vpb4Da2-resistant colony were sufficient to continue that colony. F3 larvae from the Vpb4Da2-resistant colony were relaxed to increase population size. F4 larvae were selected on Vpb4Da2 maize, but the colony was relaxed again in F5 owing to the low number of larvae surviving to adults. After F5, the colony began to stabilize and generations F6–F12 and F14 were selected on Vpb4Da2 maize (for a total of 14 generations, of which 10 were under selection; see S2 Fig).

### Confirmation of resistance

#### Larval recovery from traited and NT plants.

A target of 50 neonate larvae (<24 h old) each of susceptible, Mpp75Aa1.1-resistant, and Vpb4Da2-resistant WCR were infested into trays containing seedling maize (trays prepared as described above). For each of the three colonies, larvae were infested into two trays each of NT, Mpp75Aa1.1, and Vpb4Da2 maize. All trays (3 colonies × 3 maize types × 2 trays) were incubated at 23.8°C for 10 days. The resulting root mats were then placed in Berlese funnels for 3 days. Larvae falling to the bottom of the funnels were collected in small tubes containing ethanol and counted. This experiment was performed twice.

#### Beetle emergence from traited and NT plants.

A target of 50 neonate larvae (<24 h old) each of susceptible, Mpp75Aa1.1-resistant, and Vpb4Da2-resistant WCR were infested into trays containing seedling maize as described above for larval recovery. For each of the three colonies, larvae were infested into two trays each of NT, Mpp75Aa1.1, and Vpb4Da2 maize. After 15 days, root mats were placed onto new root mats of the same maize type for an additional 9 days and placed without lids into beetle emergence cages, where the larvae were allowed to pupate. Emerged beetles were collected and counted 3 days per week until no beetles had emerged for 2 weeks. This experiment was performed twice.

### Resistance characterization and dominance based on protein bioassays

#### Insects.

After extensive selection, the Mpp75Aa1.1- and Vpb4Da2-resistant WCR colonies were presumed to be effectively homozygous resistant (denoted as RR) to the indicated maize type, and the susceptible colony was presumed to be homozygous susceptible (denoted as SS). In addition, two heterozygous colonies were created for each protein. Approximately 150 Mpp75Aa1.1-resistant males (F13) and Vpb4Da2-resistant males (F14) were each crossed with 150 susceptible females at the Bayer CropScience insectary (Waterman, IL) to produce heterozygotes (denoted as SR). Similarly, for the corresponding reciprocal crosses, 150 females of the appropriate resistant colony were crossed with 150 susceptible males to generate heterozygotes (denoted as RS). For all seven colonies (two RR colonies, four heterozygous populations, and the SS colony), eggs were collected and oviposition dishes replaced twice per week over the course of approximately 4 weeks. Eggs were stored at approximately 12°C until shipping to Bayer CropScience (Chesterfield, MO, USA), where they were stored or used for assay initiation.

#### Test and control substances.

*Escherichia coli*-produced Mpp75Aa1.1 (Lot# 7536),Vpb4Da2 (Lot# 7941) proteins,10 mM sodium carbonate/bicarbonate (pH 10.0) and 20 mM sodium carbonate/ bicarbonate (pH 10.0) storage buffers, respectively, were produced at Bayer CropScience, Chesterfield, MO, and kept frozen until assay initiation. Negative control substances for Mpp75Aa1.1 and Vpb4Da2 were the same storage buffers used alone.

#### Concentration–response assays.

F1 WCR larvae from each of the three homozygous colonies (Mpp75Aa1.1 RR, Vpb4Da2 RR, and SS) and F1 larvae from each of the four reciprocal crosses were used to initiate concentration–response diet-overlay bioassays to assess dominance of resistance for Mpp75Aa1.1 and Vpb4Da2. For Mpp75Aa1.1 resistance, Mpp75Aa1.1 RR, SR, and RS colonies and the SS colony were evaluated at 0, 6.3,12.5, 25, 50, 100, 200, and 400 µg Mpp75Aa1.1 per mL. For Vpb4Da2 resistance, Vpb4Da2 RR, SR, and RS colonies and the SS colony were evaluated at 0, 28.2, 56.3, 112.5, 225, 450, 900, and 1800 µg Vpb4Da2 per mL. Three replicates were conducted for both proteins. Prepared WCR diet (Frontier Scientific) was dispensed at 200 µL per well into sixteen 96-well plates (four plates × four colonies) each for Mpp75Aa1.1 and Vpb4Da2 and air-dried. Treatment solution (20 µL diluted protein or buffer only [control]) was then overlaid in each well. Each concentration per colony consisted of one column of eight wells in each of four plates, for a total of 32 larvae were tested per concentration per colony per replicate. Plates were infested with one larva (<24 hr old) per well. Plates were sealed and incubated for 7 days in an environmental chamber at 27°C. At the end of the 7-day period, the larval mortality was recorded. (colony × test substance × concentration). Mean control (buffer-only) mortality did not exceed 13% for any assay (colony × protein) in any experimental replicate. The resistance ratio for Mpp75Aa1.1-resistant WCR was determined by dividing the LC_50_ of the resistant colony by that of the susceptible colony. The LC_50_ for the Vpb4Da2-resistant colony could not be determined because there was no concentration response (i.e., no lethality) at the highest concentration tested, so a resistance ratio could not be calculated, but a lower bound was estimated.

### Statistical analysis

All calculations were performed in R version 4.5.0 [15].

#### Resistance determination.

The response variable is the total number of larvae recovered, or the number of beetles emerged, summed over all collections. A mixed model was not used because two replicates and two trays per colony × treatment combination were insufficient to estimate variances.

Colony and treatment were combined into a single factor, coltrt, because the design is not a full 2-way factorial. The full model for both larval recovery and adult emergence experimental designs was


$$\begin{array}{c} \begin{array}{cc} og\left(\frac{p}{\text{1}-p}\right)= & coltrt+coltrt:Replicate/Tray\\ Y\sim & Binom(n,p) \end{array} \end{array}$$

where Y is the number of larvae or beetles recovered, *p* is the probability of an individual surviving to recovery or emergence, and *n* is the number of larvae infested into a tray at the beginning of the experiment. Replicates werenested in the treatment × colony interaction and Tray was nested in the replicate × colony × treatment interaction. However, this model fails in the present case because one or more trays with the susceptible colony feeding on Mpp75Aa1.1 had no survivors, leading to biased maximum likelihood estimates [16]. Inspection of plots of marginal means confirmed that ignoring variation between trays would not alter the comparisons between treatments and colonies, so the response was summed over trays and the simpler 3-way interaction model of treatment × colony × replicate was fitted. For larval recovery, this was sufficient to solve complete separation. For beetle emergence, the simpler 3-way model still showed signs of complete separation, so the penalized likelihood model of Firth [16] as implemented in Heinze et al. [17] was used to fit the 3-way interaction model. This approach retains the same binomial logistic-regression model, but uses Firth’s penalized likelihood to obtain finite parameter estimates when complete separation would otherwise produce infinite maximum-likelihood estimates. All pairwise comparisons of treatment and colony averaged over replicates were calculated using Lenth and Piaskowski [18], adjusting *p*-values with Tukey’s Honestly Significant Difference (HSD) test. Six planned contrasts were tested with multivariate *t* correction for multiplicity (S1 and S2 Tables):

1. For each resistant colony, performance on NT against performance of the susceptible colony on NT, hypothesized to be not significantly different (2 contrasts)
2. For each resistant colony, performance on NT against performance on the corresponding trait (used to estimate dose; 2 contrasts)
3. For the susceptible colony, performance on NT against performance on each trait (used to estimate dose; 2 contrasts)

#### Dominance estimation.

Dominance estimates are on a scale from 0 to 1, where 0 is fully recessive and 1 is fully dominant. For a dose–response assay, dominance (D) can be estimated separately for each tested concentration, depending on the mortality level (ML) of each colony at each concentration. This ML-dependent dominance, denoted D_ML_, typically decreases as mortality level increases:


$$\begin{array}{c} D_{ML}=\frac{ML_{RS}-ML_{SS}}{ML_{RR}-ML_{SS}} \end{array}$$

(In this equation, ML_RS_ represents the average of the ML estimates for the reciprocal crosses [RS and SR]). Effective dominance, here denoted D_EFF_, is the dominance estimate corresponding to the field-level dose for a particular traited event. This can be estimated from the dose–response data described above by interpolation between tested concentration levels. In the case of Mpp75Aa1.1 and Vpb4Da2, dose (percent reduction in WCR beetle emergence on traited maize compared to NT maize) was previously estimated for single-protein events tested at two field locations as 99.15% (standard deviation [SD]: 0.495) and 97.6% (SD: 0.495) for Mpp75Aa1.1 and Vpb4Da2, respectively [4,5]. To estimate D_EFF_ for these events (as an indication of what should be expected from *in planta* use of these proteins), probit analyses were conducted separately for each colony. Given fitted probit curves, dominance was computed as a function of concentration and then paired with the SS probit curve to obtain a dominance–mortality curve, which shows dominance as a function of SS mortality and allows for dominance to be estimated at any SS mortality level. Confidence bands for the dominance–mortality curve was estimated using the parametric bootstrap, as follows:

1. For each colony, the fitted curve was used to simulate 10,000 datasets using the parametric bootstrap. Each bootstrap simulation produces a dataset the same size as the original dataset.
2. For each simulated dataset, probit analyses were conducted and dominance curves computed. Quantile regression was then applied to predicted paired values (dominance, mortality) across all simulations to obtain confidence bands for the region of interest (SS mortality), focusing in this case on mortality levels ranging from 0.90 to 1.0. Basis splines were used to accommodate the non-linearity of these curves (see S3 Fig).

Because dominance is dose-dependent, D_EFF_ is the most flexible and appropriate measure of dominance [19] to assess resistance risk because it can be adapted to field-based estimates of the dose to which pests are exposed.

## Results

### Characterizing WCR populations resistant to maize expressing Mpp75Aa1.1 or Vpb4Da2

#### Larval recovery from traited plants.

Larval recovery from both resistant colonies was not significantly different from that of susceptible WCR when both were tested on NT plants (Fig 1, S1 Table). However, the recovery of susceptible insects on either Mpp75Aa1.1 or Vpb4Da2 maize was significantly less (*p* < 0.05) than recovery on NT maize. In contrast, recovery of Mpp75Aa1.1- or VpbrDa2-resistant insects on NT was not significantly different from that on their respective traited maize line (Fig 1, S1 Table).

**Fig 1:**
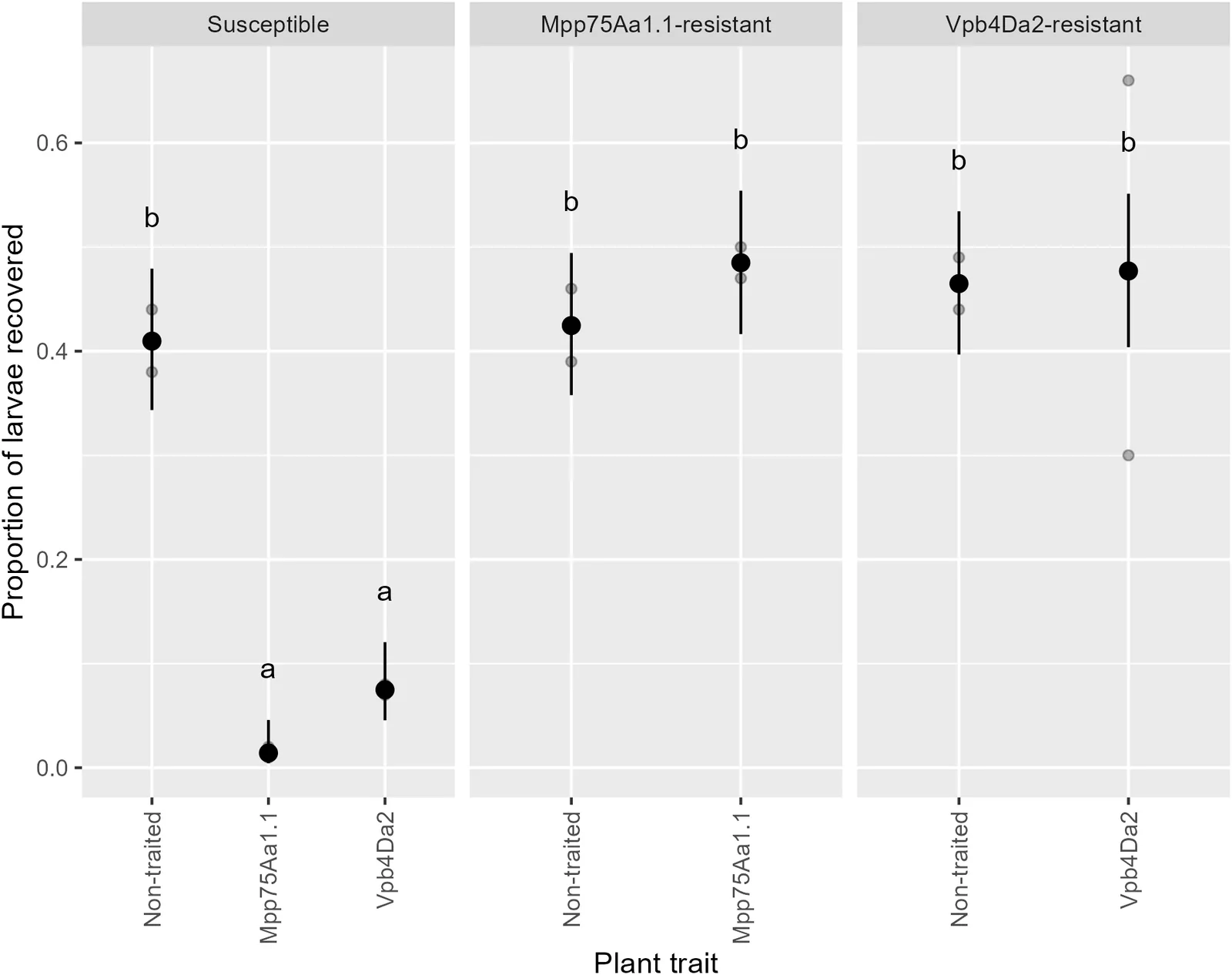
Proportion of larvae infested that were subsequently recovered from susceptible and Vpb4Da2- and Mpp75Aa1.1-resistant WCR on non-traited, Vpb4Da2, and Mpp75Aa1.1 maize averaged over two replicates. Error bars: 95% confidence intervals. Points with the same letter are not significantly different using Tukey’s HSD adjustment for a family of seven estimates. Small gray points are the raw observed fractions from the two replicates.

#### Beetle emergence from traited plants.

Susceptible insects had significantly (*p* < 0.05) higher beetle emergence on NT maize than either of the resistant colonies, but had significantly lower beetle emergence on either Mpp75Aa1.1 or Vpb4Da2 maize than did either resistant colony on any type of maize (Fig 2, S2 Table). There were no significant (*p* < 0.05) differences between Mpp75Aa1.1-resistant insects on NT and Mpp75Aa1.1 maize or between Vpb4Da2-resistant insects on NT and Vpb4Da2 maize (Fig 2, S2 Table).

**Fig 2:**
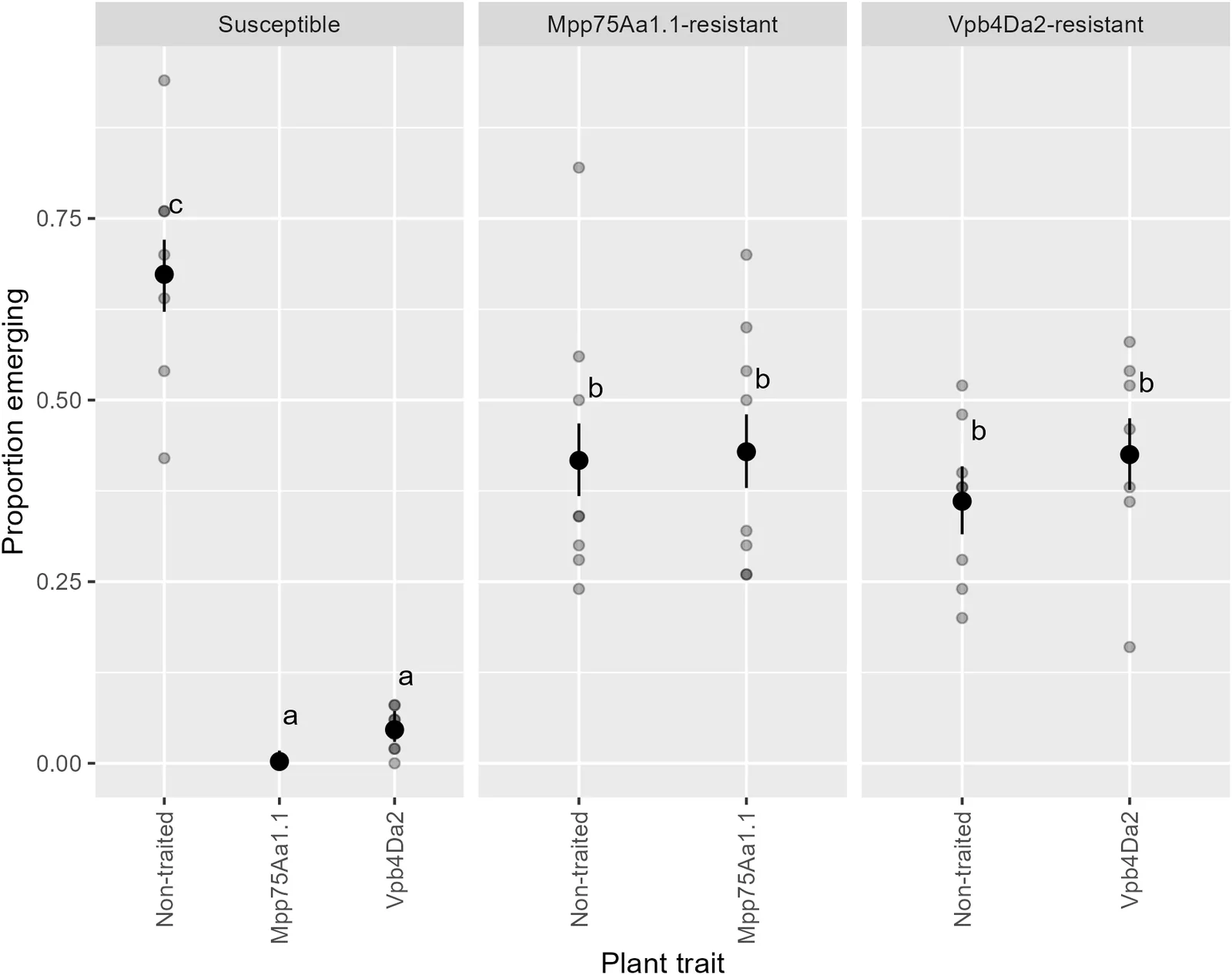
Proportion of larvae infested that emerged as beetles from susceptible and Vpb4Da2- and Mpp75Aa1.1-resistant WCR on non-traited, Vpb4Da2, and Mpp75Aa1.1 maize averaged over two replicates. Error bars: 95% confidence intervals. Points with the same letter are not significantly different using Tukey’s HSD adjustment for a family of seven estimates. Small gray points are the raw observed fractions from the two replicates.

### Response of Mpp75Aa1.1-resistant colony crosses to Mpp75Aa1.1 protein

The estimated LC_50_ values and corresponding 95% confidence intervals (CIs) for the Mpp75Aa1.1-resistant and susceptible WCR exposed to Mpp75Aa1.1 were 158.4 (137.3–182.8) and 30.6 (27.2–34.5) µg Mpp75Aa1.1/mL diet, respectively (Table 1). Reciprocal crosses (SR and RS) exhibited concentration responses between those of the RR and SS crosses, with LC_50_ values of 73.0 (64.9–82.1) and 63.1 (55.6–71.6) µg Mpp75Aa1.1/mL diet, respectively, and were not significantly different (based on overlap of 95% CI), demonstrating that resistance was not sex-linked (Table 1).

**Table 1:** Response of Mpp75Aa1.1-susceptible, Mpp75Aa1.1-resistant, and reciprocal crosses of WCR to Mpp75Aa1.1 protein in bioassays.

Colony	N^a^	Slope	SE^b^	LC_50_^c^	95% CI^d^	χ^2^	df^e^	*p*-Value
SS	664	1.18	0.08	30.6	27.2–34.5	75.7	80	0.61
SR	667	1.18	0.08	73.0	64.9–82.1	78.8	80	0.52
RS	664	1.04	0.07	63.1	55.6–71.6	69.5	80	0.42
RR	668	1	0.08	158.4	137.3–182.8	81.2	80	0.44

^a^Total number of insects tested across all non-zero concentrations. Three replicates (target of 32 insects/replicate) per concentration.

^b^Standard error.

^c^LC_50_ values based on seven concentrations. Values indicate µg Mpp75Aa1.1 protein/mL diet.

^d^95% confidence interval.

^e^Degrees of freedom.

### Response of Vpb4Da2-resistant colony crosses to Vpb4Da2 protein

An LC_50_ value could not be estimated for the Vpb4Da2-resistant colony cross (RR) because there was no concentration response even at the highest concentration tested. Susceptible WCR (SS) exposed to Vpb4Da2 had an LC_50_ value and corresponding 95% CI of 169.7 (150.7–191.2) µg Vpb4Da2/mL diet (Table 2). Reciprocal crosses (SR and RS) exhibited concentration responses between those of the RR and SS crosses, with LC_50_ values of 427.0 (371.7–490.5) and 446.4 (385.1–517.4) µg Vpb4Da2/mL diet, respectively, and were not significantly different (based on overlap of 95% CI), demonstrating that resistance was not sex-linked (Table 2).

**Table 2:** Response of Vpb4Da2-susceptible, Vpb4Da2-resistant, and reciprocal crosses of WCR to Vpb4Da2 protein in bioassays.

Colony	N^a^	Slope	SE^b^	LC_50_^c^	95% CI^d^	χ^2^	df^e^	*p*-Value
SS	660	1.16	0.08	169.7	150.7–191.2	69.53	80	0.79
SR	664	0.94	0.06	427.0	371.7–490.5	83.08	80	0.38
RS	663	0.86	0.06	446.4	385.1–517.4	69.53	80	0.97
RR	671	–	–	>1800^f^	–	–	–	–

^a^Total number of insects tested across all non-zero concentrations. Three replicates (target of 32 insects/replicate) per concentration.

^b^Standard error.

^c^LC_50_ values based on seven concentrations. Values indicate µg Vpb4Da2 protein/mL diet.

^d^95% confidence interval.

^e^Degrees of freedom.

^f^1800 µg Vpb4Da2 protein/mL diet represents the highest concentration tested.

### Dominance

#### Dominance of Mpp75Aa1.1.

Dominance values for SS mortality at all concentrations evaluated in the Mpp75Aa1.1 concentration–response study declined from 0.71 (partially dominant) at 4% mortality to 0.09 (largely recessive) at 99% mortality (S3 Table). Fig 3 shows dominance as a function of SS mortality estimated from the original data, as well as 95% confidence bands (dashed lines) estimated from 10,000 parametric bootstrap simulations. At the previously estimated field-level dose for a single-protein event of 99.15%, dominance was estimated as D_EFF_ = 0.23 (95% CI 0.12–0.35).

**Fig 3:**
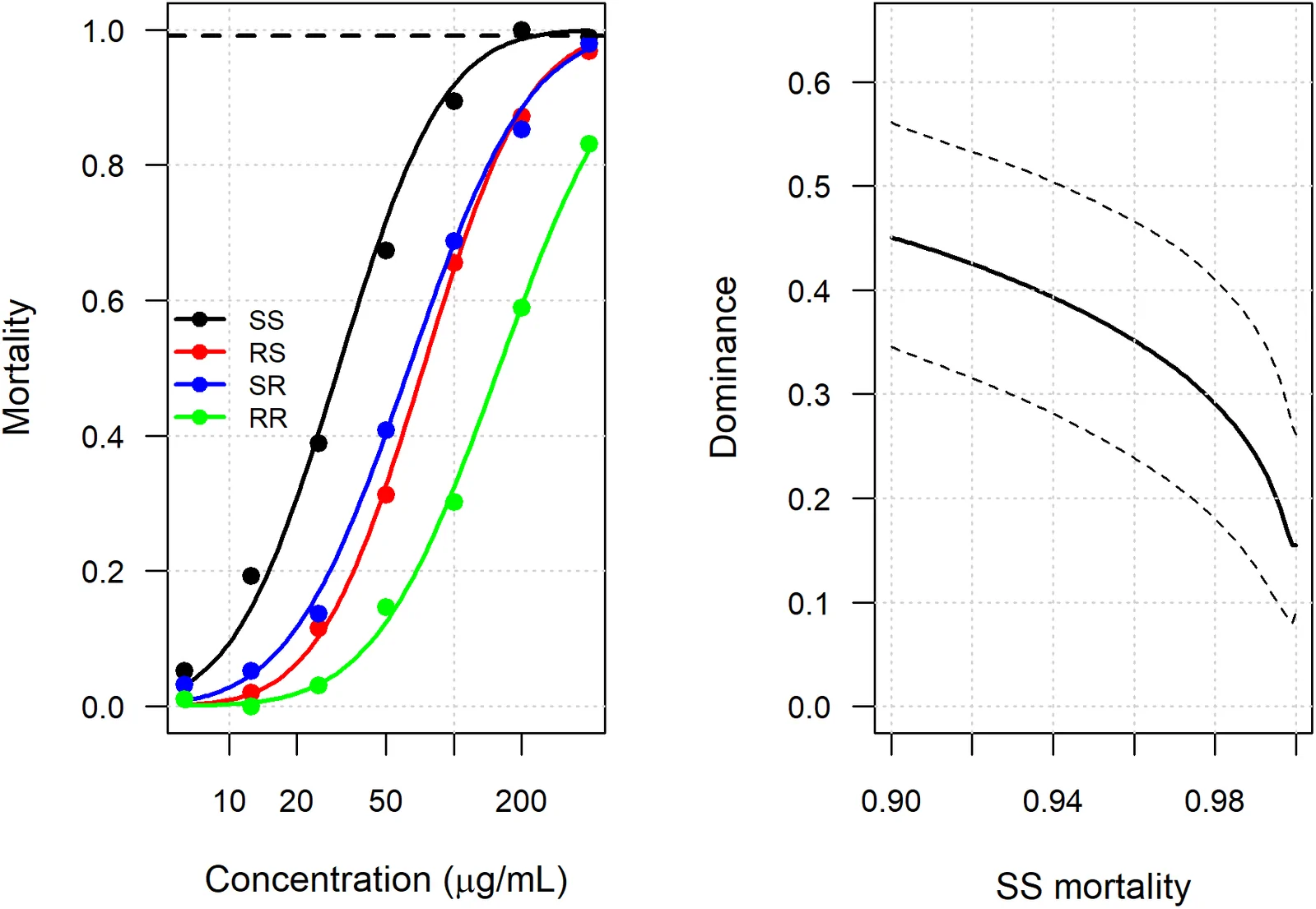
Concentration–response curves for all colonies (left, excluding zero-concentration mortality) and dominance–mortality curve in the SS (susceptible colony) mortality range [0.90, 1.0] with 95% confidence bands (right) for Mpp75Aa1.1. Dashed black line in concentration–response figure corresponds to an estimated field-level dose of 99.15%. At that dose, D_EFF_ is estimated to be 0.23 (95% CI 0.12–0.35). RR is the Mpp75Aa1.1-resistant colony; RS and SR are heterozygous colonies obtained from reciprocal RR × SS crosses.

#### Dominance of Vpb4Da2.

Dominance values for SS mortality at all concentrations evaluated in the Vpb4Da2 concentration–response study declined from 0.83 (partially dominant) at 3% mortality to 0.07 (largely recessive) at 100% mortality (S4 Table). Fig 4 shows dominance as a function of SS mortality estimated from the original data, as well as 95% confidence bands (dashed lines) estimated from 10,000 parametric bootstrap simulations. At the previously estimated field-level dose for a single-protein event of 97.6%, dominance was estimated as D_EFF_ = 0.24 (95% CI 0.16–0.32).

**Fig 4:**
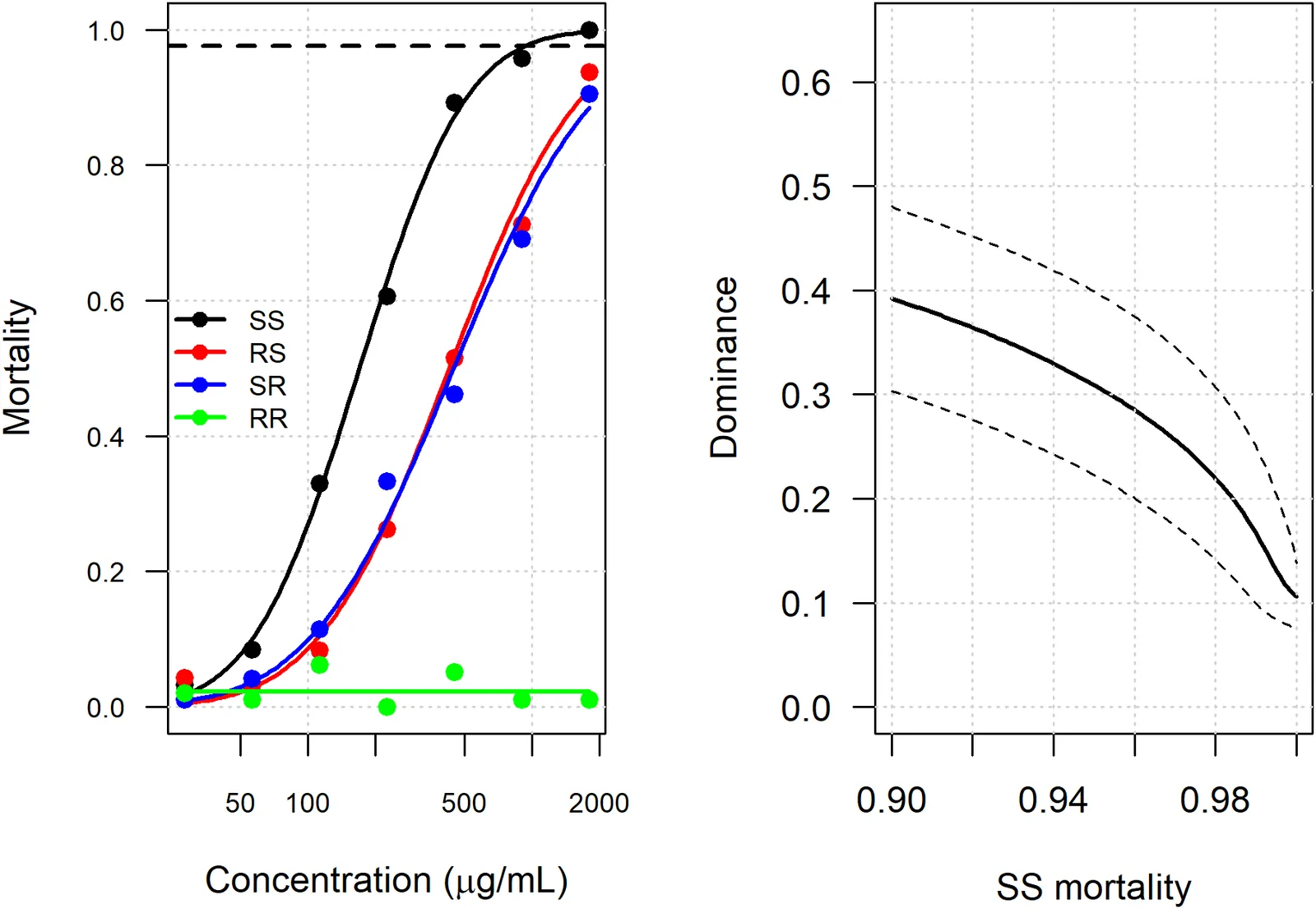
Concentration–response curves for all colonies (left, excluding 0-concentration mortality) and dominance–mortality curve in the SS (susceptible colony) mortality range [0.90, 1.0] with 95% confidence bands (right) for Vpb4Da2. Dashed black line in concentration–response figure corresponds to an estimated field-level dose of 97.6%. At that dose, D_EFF_ is estimated to be 0.24 (95% CI 0.16–0.32). RR is the Vpb4Da2-resistant colony; RS and SR are heterozygous colonies obtained from reciprocal RR × SS crosses.

## Discussion

Almost all insecticidal protectants that have been expressed *in planta* in commercial products thus far have been 3-domain crystalline (Cry) or Vip3 proteins derived from Bt, so almost all reported cases of field-relevant insect resistance to *in planta* toxins have involved such proteins [20,21]; one exception is resistance to DvSnf7 dsRNA in WCR [14]. Based on these cases, insect resistance has been typified as conferred by a single major gene, autosomal, partially to fully recessive, and largely receptor-mediated [21]. In this study, resistance to the Mpp75Aa1.1 and Vpb4Da2 proteins was phenotypically similar to these previously described cases of resistance to Cry and Vip3 proteins. In particular, the autosomal nature and partially recessive level of dominance were comparable to previous reports of WCR resistance to *in planta* Bt proteins [22,23]. Here, effective dominance was estimated to be 0.23 and 0.24 for Mpp75Aa1.1 and Vpb4Da2, respectively, based on a previous assessment of dose for single-protein events with these proteins [4,5]. More generally, dominance was estimated to vary from 0.45 to 0.15 for MppAa1.1 resistance and between 0.40 and 0.10 for Vpb4Da2 resistance for doses between 0.9 and 1.0. Previous dominance estimates for WCR on Cry3Bb1-containing plant tissue averaged 0.34 (four field-collected populations, range 0.08–0.54) [22] and 0.36 on Gpp34/Tpp35Ab1-containing plant tissue [23]. In these previous studies, WCR were tested on plant material in the laboratory, whereas the present estimates of dominance were derived from diet bioassay results and then compared to a field-relevant dose. As in the current study, Smith et al. [23] used a susceptible colony derived from the same field collection as the resistant strain and backcrossed the resistant colony with the susceptible colony throughout the selection process to maximize the similarity in the genetic background. In contrast, Shrestha and Gassmann [22] used a susceptible colony that had been maintained in the laboratory for many years prior to crossing with the resistant colony. It is worth noting that the D_EFF_ estimates for DvSnf7 dsRNA resistance in WCR [14] are lower than the estimates in this and previous studies for bacterially derived proteins. This difference is in keeping with the evidence that resistance to DvSnf7 dsRNA, which acts through an RNAi mechanism, was a function of its uptake [14] and thus caused by a fundamentally different mechanism than seems to be common for bacterial proteins.

The similarity between the characteristics of the WCR resistance to the Mpp75Aa1.1 and Vpb4Da2 proteins described here and previous descriptions of WCR resistance to Cry Bt proteins suggests that the risk of resistance evolving to these proteins will be comparable to existing technologies for WCR control (i.e., the resistance risk will be high, all other things being equal). Consequently, these new proteins will need to be pyramided with additional modes of action, such as a dsRNA-based technology, to reduce this resistance risk. It also will be important to continue to encourage the use of additional tactics for CRW control and resistance management, such as crop rotation, when such technologies are introduced.

The generations of selection needed to develop a resistant colony can be affected by many factors including initial resistance allele frequency and number of insects under selection, selection regime (dose and method of selection), and fitness costs associated with the mechanism of resistance. At least one of these factors (e.g., the dose for Mpp75Aa1.1 maize being considerably higher than that for Vpb4Da2) may explain the initial die-off of the Mpp75Aa1.1-resistant colony (using males from the field) after the initial round of selection in the lab. As there are few WCR-active traits commercialized, there are few published reports with which to determine whether the 10 generations of selection used to develop resistance to Mpp75Aa1.1 and Vpb4Da2 maize in WCR is typical [24]. As in Khajuria et al. [14], initial resistance selection in the present study occurred in the field using >3000 plants per trait under relatively high WCR pressure. This method not only increased the likelihood of capturing resistance alleles but also ensured that the dose for both traits was commercially relevant. Khajuria et al. [14] also reported that observable resistance to DvSnf7 dsRNA-expressing plants required 7–11 generations to develop, for a final resistance ratio of >130-fold. Zhao et al. [25] reported a 97-fold level of resistance to a relatively high and non-commercial level of mCry3A-containing maize (99.7% dose) by WCR after only six generations of selection, although this dose was higher than that used in the current study, especially for Vpb4Da2.

Susceptible larvae were more susceptible to Mpp75Aa1.1 protein than to Vpb4Da2 protein, consistent with previously reported beetle emergence data [4,5]. Both resistant colonies survived as well on their respective trait as on NT maize. Additionally, early larval stadia of both resistant colonies survived on NT as well as susceptible larvae, suggesting no fitness costs. In contrast, although similar numbers of beetles emerged from the resistant colonies on their respective traited plants and on NT maize (demonstrating resistance), both resistant colonies had significantly fewer beetles emerging on NT than did the susceptible colony, suggesting possible fitness costs. Comparison of larval recovery results with beetle emergence results suggests either that there are subtle differences between resistant and susceptible larvae that were not detected in the larval recovery assay or that fitness costs occur mainly at the pupal stage.

The resistance ratios for Mpp75Aa1.1- and Vpb4Da2-resistant WCR were determined using diet bioassays. As similar proportions of Mpp75Aa1.1-resistant WCR were recovered from their respective traited plants and from NT plants, indicating complete or near-complete resistance, a relatively high resistance ratio would be expected. However, in the diet bioassay Mpp75Aa1.1-resistant WCR exhibited a concentration response and an LC_50_ value only 5-fold that of susceptible WCR (resistance ratio = 5). This result suggests either incomplete resistance (although this would be inconsistent with the plant results) or that Mpp75Aa1.1-resistant WCR react differently when feeding on Mpp75Aa1.1-expressing plants than on Mpp75Aa1.1-treated diet. As there are no definitive data for Bt resistance mechanisms in WCR [24], interpreting possible differences in resistance mechanisms between plant- and diet-based bioassays is difficult. If Mpp75Aa1.1 protein is not uniformly distributed in maize roots, larval avoidance could help explain response differences on plants as compared to diet bioassays. In contrast, Vpb4Da2-resistant WCR had no concentration response in diet bioassays, even at the highest concentration tested (which produced 100% mortality in susceptible WCR); this finding is consistent with the larval recovery *in planta* and suggests a relatively high level of resistance. Dividing the highest concentration tested (1800 µg Vpb4Da2 protein/mL diet) by the LC_50_ of the susceptible colony (150.7) gives a lower bound for the resistance ratio of 11.9, but the actual level of resistance is likely to be 50–100-fold (WM et al., unpublished bioassay data).

## Conclusions

Bayer has developed WCR colonies resistant to Mpp75Aa1.1- and Vpb4Da2-containing maize using initial field selection and subsequent lab selection on traited plants. Resistance was phenotypically similar between the two colonies and to previous reports of WCR resistance to *in planta* insecticidal proteins, with no sex-linkage, incompletely recessive inheritance, and suggestions of fitness costs. These colonies will be a useful tool for understanding the risk of WCR resistance arising to these proteins when they are deployed commercially in traited maize.

## Supporting information

S1 Fig Mpp75Aa1.1 resistance selection process. NT, non-traited.
(TIF)

S2 Fig Vpb4Da2 resistance selection process. (TIF)

S3 Fig Approach to estimating dominance as a function of SS (susceptible colony) mortality. Effective dominance (D_EFF_) of resistance to a trait is obtained by estimating the degree of dominance at the level of SS mortality corresponding to the field-level dose of that trait. 1) Probit model is fitted for each colony; 2) parametric bootstrap is used to simulate 10,000 data sets the same size as the original data set; 3) dominance is plotted against SS mortality; 4) confidence intervals are determined using quantile regression with basis splines to accommodate the non-linear nature of the curves. RR is a resistant colony; RS and SR are heterozygous colonies obtained from reciprocal RR × SS crosses.
(TIF)

S1 Table Larval recovery planned contrasts. (DOCX)

S2 Table Adult emergence planned contrasts. (DOCX)

S3 Table Estimated SS mortality and D_ML_ and their corresponding 95% confidence intervals for each Mpp75Aa1.1 concentration tested. (DOCX)

S4 Table Estimated SS mortality and D_ML_ and their corresponding 95% confidence intervals for each Vpb4Da2 concentration tested. (DOCX)
