# Supplementary material for: Development and characterization of western corn rootworm (Diabrotica virgifera virgifera LeConte) resistant to non-Cry insecticidal proteins Mpp75Aa1.1 and Vpb4Da2
Source: PLoS One. 2026 Nov 2;21(11):e0359428. doi: 10.1371/journal.pone.0359428 (PMC13726953; doi:10.1371/journal.pone.0359428)
Supplement: Supplementary file 1 [file pone.0359428.s004.docx]

**S1 Table. Larval recovery planned contrasts.**

| **Contrast** | **Estimate** | **SE** | **Z-Ratio** | ***p*-Value** |
| --- | --- | --- | --- | --- |
| Mpp75Aa1.1-resistant on NT vs.  Susceptible on NT | 0.06 | 0.20 | 0.30 | 1.00 |
| VpbDa2-resistant on NT vs.  Susceptible on NT | 0.22 | 0.20 | 1.11 | 0.81 |
| Susceptible on Mpp75Aa1.1 vs.  Susceptible on NT | −3.88 | 0.63 | –6.13 | 0.00 |
| Susceptible on VpbDa2 vs.  Susceptible on NT | −2.15 | 0.31 | –7.04 | 0.00 |
| Mpp75Aa1.1-resistant on Mpp75Aa1.1 vs.  Mpp75Aa1.1-resistant on NT | 0.24 | 0.20 | 1.21 | 0.74 |
| VpbDa2-resistant on VpbDa2 vs.  VpbDa2-resistant on NT | 0.05 | 0.21 | 0.23 | 1.00 |

The estimate is the change in log-odds of larval recovery from the 2^nd^ treatment listed to the 1^st^ treatment listed.

NT, non-traited maize; SE, standard error.
