# Supplementary material for: Development and characterization of western corn rootworm (Diabrotica virgifera virgifera LeConte) resistant to non-Cry insecticidal proteins Mpp75Aa1.1 and Vpb4Da2
Source: PLoS One. 2026 Nov 2;21(11):e0359428. doi: 10.1371/journal.pone.0359428 (PMC13726953; doi:10.1371/journal.pone.0359428)
Supplement: Supplementary file 2 [file pone.0359428.s005.docx]

## S2 Table. Adult emergence planned contrasts.

| **Contrast** | **Estimate** | **SE** | **Z-Ratio** | ***p*-Value** |
| --- | --- | --- | --- | --- |
| Mpp75Aa1.1-resistant on NT vs.  Susceptible on NT | –1.06 | 0.16 | –6.77 | 0.00 |
| VpbDa2-resistant on NT vs.  Susceptible on NT | –1.29 | 0.16 | –8.34 | 0.00 |
| Susceptible on Mpp75Aa1.1 vs.  Susceptible on NT | –6.72 | 1.01 | –6.66 | 0.00 |
| Susceptible on VpbDa2 vs.  Susceptible on NT | –3.75 | 0.27 | –14.07 | 0.00 |
| Mpp75Aa1.1-resistant on Mpp75Aa1.1 vs.  Mpp75Aa1.1-resistant on NT | 0.05 | 0.15 | 0.33 | 1.00 |
| VpbDa2-resistant on VpbDa2 vs.  VpbDa2-resistant on NT | 0.27 | 0.15 | 1.85 | 0.31 |

The estimate is the change in log-odds of adult emergence from the 2^nd^ treatment listed to the 1^st^ treatment listed.

NT, non-traited maize; SE, standard error.
