# Supplementary material for: Development and characterization of western corn rootworm (Diabrotica virgifera virgifera LeConte) resistant to non-Cry insecticidal proteins Mpp75Aa1.1 and Vpb4Da2
Source: PLoS One. 2026 Nov 2;21(11):e0359428. doi: 10.1371/journal.pone.0359428 (PMC13726953; doi:10.1371/journal.pone.0359428)
Supplement: Supplementary file 3 [file pone.0359428.s006.docx]

**S3 Table. Estimated SS (susceptible colony) mortality and D_ML_ and their corresponding 95% confidence intervals for each Mpp75Aa1.1 concentration tested.**

|  | **SS mortality** | | | **D_ML_** | | |
| --- | --- | --- | --- | --- | --- | --- |
| **Concentration (µg/mL)** | **Est** | **LCI** | **UCI** | **Est** | **LCI** | **UCI** |
| 6.3 | 0.04 | 0.01 | 0.08 | 0.71 | 0.05 | 2.76 |
| 12.5 | 0.15 | 0.09 | 0.24 | 0.8 | 0.64 | 0.94 |
| 25 | 0.37 | 0.27 | 0.48 | 0.77 | 0.63 | 0.9 |
| 50 | 0.71 | 0.6 | 0.8 | 0.62 | 0.49 | 0.77 |
| 100 | 0.92 | 0.85 | 0.96 | 0.36 | 0.24 | 0.5 |
| 200 | 1 | 0.97 | 1 | 0.3 | 0.18 | 0.45 |
| 400 | 0.99 | 0.97 | 1 | 0.09 | −0.02 | 0.28 |

D_ML_ indicates the degree of dominance at each mortality level. LCI and UCI represent the lower and upper bound, respectively, of each 95% confidence interval.
