# Supplementary material for: Development and characterization of western corn rootworm (Diabrotica virgifera virgifera LeConte) resistant to non-Cry insecticidal proteins Mpp75Aa1.1 and Vpb4Da2
Source: PLoS One. 2026 Nov 2;21(11):e0359428. doi: 10.1371/journal.pone.0359428 (PMC13726953; doi:10.1371/journal.pone.0359428)
Supplement: Supplementary file 4 [file pone.0359428.s007.docx]

**S4 Table. Estimated SS (susceptible colony) mortality and D_ML_ and their corresponding 95% confidence intervals for each Vpb4Da2 concentration tested.**

|  | **SS mortality** | | | **D_ML_** | | |
| --- | --- | --- | --- | --- | --- | --- |
| **Concentration (µg/mL)** | **Est** | **LCI** | **UCI** | **Est** | **LCI** | **UCI** |
| 28.2 | 0.03 | 0.01 | 0.08 | 0.83 | −7.6 | 12.26 |
| 56.3 | 0.08 | 0.04 | 0.14 | 0.69 | 0.31 | 1.12 |
| 112.5 | 0.32 | 0.23 | 0.42 | 0.89 | 0.69 | 1.14 |
| 225 | 0.61 | 0.51 | 0.71 | 0.54 | 0.43 | 0.65 |
| 450 | 0.9 | 0.83 | 0.95 | 0.48 | 0.39 | 0.58 |
| 900 | 0.96 | 0.91 | 0.99 | 0.26 | 0.19 | 0.33 |
| 1800 | 1 | 0.97 | 1 | 0.07 | 0.04 | 0.11 |

D_ML_ indicates the degree of dominance at each mortality level. LCI and UCI represent the lower and upper bound, respectively, of each 95% confidence interval.
